# Remembering Ken Stein

**DOI:** 10.1017/S0266462324000278

**Published:** 2024-05-10

**Authors:** Don Husereau, Karl Claxton

**Affiliations:** 1 School of Epidemiology and Public Health, University of Ottawa, Ottawa, ON, Canada; 2 Institute of Health Economics, Edmonton, AB, Canada; 3 Centre for Health Economics, University of York, York, UK.

*You’re mad. Bonkers Off your head. But I’ll tell you a secret. All the best people are. – Alice in Wonderland (2010).* (1)

Professor Ken Stein has died.

As well as being a former member of the HTAi Board of Directors (2007–11) and Editorial Board (2012–24) of this journal, he was, without a doubt, fit for the Mad Hatter’s tea party. Ken shone like the sun. He brought laughter and joy to all those fortunate enough to know and work with him. He also brought infectious energy, inquisitiveness, and open-minded enthusiasm to everything he turned his mind to.

Over his career, Ken made a significant and lasting impact to health technology assessment (HTA) in the United Kingdom and internationally. This included his early involvement in the National Institute for Health Research (NIHR) HTA program in the 1990s until his appointment as Director of the NIHR Systematic Review Programme in 2018, as well as being the founding director of NICE’s Peninsula Technology Assessment Group (PenTAG) in 2003, serving as a member and Vice Chair of one of NICE’s Appraisal Committees for 15 years, and serving as Editor-in-Chief for of the monograph series *Health Technology Assessment.*

In addition to his significant contributions to HTA, Ken also led initiatives in evidence synthesis and health services research including shaping the redesigned Cochrane Collaboration and establishing the NIHR Applied Research Collaboration for the South West Peninsula (PenARC), serving as Deputy Director and pioneering patient and public involvement initiatives in this research.

Ken graduated in medicine from the University of Bristol in 1987, and then worked as a physician in the United Kingdom and abroad before specializing in public health medicine in Southampton. In 1999, he moved to Exeter to work as a consultant in Public Health Medicine in his local health authority, and then Director of Public Health for the Primary Care Trust. He was rewarded a position of Chair in Public Health in 2007.

Ken was diagnosed with metastatic renal cancer in 2019 and went on to take medications that were considered by the very same HTA committee he once belonged to. He was fully aware of his prognosis and continued to live life to its fullest. In addition to his many accomplishments, Ken had a passion for music and had become an accomplished singer and guitar player, even playing professionally. In the final days before his death at his country home in Exeter, Ken had successfully recorded music for his own memorial – the last of a series of remarkable accomplishments.

More importantly, Ken was our good friend. He had a child-like sense of wonder, and always made us think, and laugh, out loud. He was also, by all accounts, a wonderful son, spouse, sibling, father, and grandfather. He has been described by one peer as being “unfailingly kind, courteous, wise, and generous with his patience and knowledge.”

We could not agree more.
